# Knowledge and Support Needs of School-Based Speech and Language Pathologists in Addressing Dyslexia

**DOI:** 10.3390/brainsci16050466

**Published:** 2026-04-27

**Authors:** Mazen Almethen

**Affiliations:** Department of Special Education, College of Education, Qassim University, Buraydah 52571, Saudi Arabia; m.almethen@qu.edu.sa

**Keywords:** dyslexia, school-based SLPs, knowledge, Saudi Arabia, support needs

## Abstract

**Highlights:**

**What are the main findings?**

- Most participants held misconceptions about dyslexia, rating their knowledge as very limited.
- School-based SLPs viewed their role as minimal, often considering it secondary to that of special education teachers.

**What are the implications of the main findings?**

- Assist stakeholders in understanding the current level of dyslexia-related knowledge among school-based SLPs.
- Inform stakeholders about factors to improve school-based SLPs’ practices regarding dyslexia.

**Abstract:**

**Background/Objectives**: This study aimed to investigate school-based speech–language pathologists’ (SLPs) perceptions of their ability and needs to identify and remediate students with dyslexia. **Methods**: A qualitative approach was employed, utilizing semi-structured interviews with school-based SLPs. The findings were thematically analyzed by following six steps; (a) familiarization with the data; (b) initial code generation; (c) theme identification; (d) theme review; (e) defining and naming themes; and (f) report writing. **Results**: The findings revealed that participants had a very limited knowledge of dyslexia, and most did not consider reading difficulties within their scope of practice. Professional development opportunities, collaboration with teachers, and administrative support were identified as crucial factors to enhance SLPs’ practice supporting dyslexic cases in school settings. **Conclusions**: The study showed that school-based SLPs lack the necessary knowledge to work with students with dyslexia, which aligns with results observed in other countries. This finding highlights the importance of empowering their abilities to effectively support students with dyslexia.

## 1. Introduction

Dyslexia, also known as a specific learning disability with impairment in reading, is considered the most prevalent learning disability worldwide, affecting 1 in 10 people [1]. Many individuals with dyslexia remain undiagnosed and receive little or no support or intervention. In the United States, school-age children with learning disabilities constitute 5 to 15% of the population, of whom 80% have dyslexia [2,3]. In Saudi Arabia, although comprehensive data on the exact prevalence and identification of dyslexia among students are lacking, the Saudi Press Agency (SPA, 2018) reported that approximately 500,000 dyslexic students attend more than 28,000 schools across the Kingdom’s regions [4]. According to Byrne et al. (2014), dyslexia is commonly described as having two primary types: acquired dyslexia and developmental dyslexia [5]. Acquired dyslexia results from brain injury or illness, typically occurring in adulthood after literacy skills have been established. In contrast, developmental dyslexia emerges during the process of learning to read and is not associated with the significant brain damage observed in acquired dyslexia.

While there are various definitions of dyslexia, the International Dyslexia Association (IDA, 2025) provides one of the most detailed descriptions, highlighting key aspects, potential causes, and observable characteristics [6]. The definition is as follows:

“Dyslexia is a specific learning disability characterized by difficulties in word reading and/or spelling that involve accuracy, speed, or both and vary depending on the orthography. These difficulties occur along a continuum of severity and persist even with instruction that is effective for the individual’s peers. The causes of dyslexia are complex and involve combinations of genetic, neurobiological, and environmental influences that interact throughout development. Underlying difficulties with phonological and morphological processing are common but not universal, and early oral language weaknesses often foreshadow literacy challenges. Secondary consequences include reading comprehension problems and reduced reading and writing experience that can impede growth in language, knowledge, written expression, and overall academic achievement. Psychological well-being and employment opportunities also may be affected. Although identification and targeted instruction are important at any age, language and literacy support before and during the early years of education is particularly effective”.

(IDA, 2025, para. 1 [6])

Neuroscience has significantly advanced the understanding of dyslexia and supported current theories by providing evidence that individuals with dyslexia experience difficulties in language processing, including challenges with phonological processing (manipulating the sound structure of spoken language) and orthographic word processing (recognizing the visual structure of words) [7,8]. By contrast, it is important to differentiate dyslexia from other causes of poor reading performance to ensure an accurate diagnosis and appropriate support. Some children experience difficulties due to insufficient instruction or limited access to language and printed materials. In these cases, reading problems are typically associated with broader educational or language disadvantages rather than a specific cognitive impairment. These issues generally improve with focused, high-quality instruction [9].

Dyslexia can significantly impact a student’s reading and writing abilities. Without these essential language skills, students with dyslexia may face challenges in their education, social interactions, and overall well-being [10]. Early identification and appropriate interventions are crucial for helping individuals with dyslexia manage these difficulties [11,12]. When suitable support and interventions are not provided, individuals with dyslexia are less likely to succeed in their educational journey [13]. The challenges associated with dyslexia extend beyond educational experiences; research has linked dyslexia to an increased risk of mental health issues, such as anxiety and depression [14,15].

While early diagnosis and intervention are recognized as crucial factors in helping students with dyslexia reach their full potential, the actual practice of diagnosing dyslexia often falls short. Most students with dyslexia are not identified until third grade or later, and some remain undiagnosed into adolescence or adulthood [16]. In Saudi Arabia, SPA (2018) indicated that only 5% of students with dyslexia receive special education services in public schools [4]. This low percentage may be attributed to a lack of knowledge about dyslexia among education professionals. Research indicates that educators often lack the necessary preparation and training to effectively identify the signs and symptoms of dyslexia in their students [17,18]. Therefore, it is emphasized that school professionals’ awareness and recognition of the key indicators of dyslexia are essential for the early identification of students with this learning disability.

School-based speech–language pathologists (SLPs) are among the professionals mentioned in the recommended assessment and intervention protocols for students with dyslexia, as they are often the first to recognize language development concerns in these students [19]. In 2001, the American Speech–Language–Hearing Association (ASHA) released a position statement affirming that SLPs are key players in identifying, diagnosing, and supporting students with dyslexia due to their expertise in understanding the unique language challenges these students face and in developing effective interventions. According to the statement, their roles include preventing, identifying, assessing, intervening, and advocating for effective literacy practices for children, including those with dyslexia. Therefore, it is critical, if not mandatory, for SLPs to be part of the multidisciplinary team involved in identifying and managing students with dyslexia [20].

According to the Saudi Ministry of Education (2025) and based on the information in the Organizing Guide for Special Education, the school-based scope of practice for SLPs includes assessing and providing treatment for students with language and speech disorders [21]. Students become eligible for special education-related services, including speech and language therapy, after being identified with one or more disabilities by a multidisciplinary team. Based on personal observation and experience, school-based SLPs were assigned either to public schools with self-contained special education classes or to special education schools to provide services to students with complex communication disorders and difficulties.

Despite the significant role of school-based SLPs in managing reading challenges in Saudi public schools, research on their knowledge of dyslexia remains limited. Farran et al. (2023) noted that while studies on dyslexia in the Arab world have primarily focused on children and adolescents with reading difficulties, there is a substantial gap in understanding how teachers and other school professionals are prepared to address this learning disorder [22]. Al-Malki and Bukari (2024), based on personal observation and experience, highlighted the lack of dyslexia-related knowledge among school-based SLPs in Saudi Arabia [23].

Researchers have emphasized the importance of education professionals possessing both a strong understanding of reading instruction and a clear grasp of reading difficulties, including dyslexia, as a key factor in effectively intervening with affected students [18,24]. Consequently, a lack of knowledge about dyslexia among school-based SLPs can negatively impact their ability to identify the condition early and provide high-quality educational services to these students. Exploring SLPs’ self-perceptions in this area can help identify gaps in their preparation and guide the development of targeted professional development opportunities. The findings of this study will contribute to the current body of knowledge regarding the level of dyslexia-related understanding among school-based SLPs. It will highlight potential gaps in their knowledge and identify areas for targeted professional development. Additionally, this research may inform best practices for supporting students with dyslexia, ultimately enhancing the effectiveness of interventions and promoting improved educational outcomes in the future.

This study aims to explore school-based SLPs’ perceptions regarding their ability to identify and remediate students with dyslexia. To achieve this aim, the study sought to answer the following research questions:

1. How do school-based SLPs perceive their personal knowledge and skills to identify and remediate students with dyslexia?
2. How do school-based SLPs perceive their roles in working with students with dyslexia?
3. What resources or support do school-based SLPs need to enhance their ability to work with students with dyslexia?

## 2. Materials and Methods

### 2.1. Research Design

The researcher employed a qualitative interviewing approach for this study because it aligns well with the study’s objectives. According to Stake (2010), qualitative research draws on individuals’ perspectives to provide meaningful interpretations of how processes function [25]. This approach offers several advantages: (a) it focuses on participants’ lived experiences; (b) it takes place in natural settings, allowing researchers to gain detailed insights into the issue; and (c) it enables researchers to obtain comprehensive findings [26,27].

### 2.2. Participants

Participants were six school-based SPLs who were selected using a purposive sampling technique and met the following criteria: (a) had worked as school-based SLPs; (b) had more than two years of experience as school-based SLPs; (c) held a certificate in speech and language pathology; and (d) were willing to participate in the study.

Participants were recruited through an invitation email disseminated to the Special Education Department within the General Directorate of Education in the Qassim region of Saudi Arabia. Initially, contact was established with the General Directorate of Education in Qassim, accompanied by the submission of requisite documentation, including the study’s objectives and the interview protocol, to secure a letter of support for the research. Upon obtaining this letter of support and receiving approval from the Institutional Review Board (IRB) at Qassim University, the invitation email was sent to the Special Education Department. Subsequently, the department facilitated communication by encouraging SLPs who expressed interest in participating to contact the researcher via email or telephone. Interested participants reached out, and I provided them with details about the interview process and informed consent.

All participants were from schools or special education centers under the General Directorate of Education in Qassim region, Saudi Arabia. Since all general directorates of education operate under the laws and regulations of the Ministry of Education, the specific school district included in the study was chosen due to its proximity to my residence and my positive relationships with the principals of schools in this area.

Among the participants, five individuals possessed a bachelor’s degree in speech and language pathology, comprising three males and two females. Additionally, one male participant held a bachelor’s degree in special education along with a diploma in speech and language pathology. All participants had more than three years of experience working as school-based SLPs in various settings. Five of them worked in general public schools that included self-contained special education classes, primarily serving students with disabilities as well as some students from general education. Four participants worked at special education centers that receive cases from all schools under the General Directorate of Education in the region. These centers typically accept students who cannot be served by their home schools. Table 1 shows the participants’ demographic characteristics.

### 2.3. Data Collection and Analysis

A semi-structured interview was developed to collect data from six school-based SLPs. I scheduled appointments with the participants at times convenient for them. All six interviews were conducted via the online voice call application Zoom. I explained the study, obtained written consent, and clarified any terminology that might cause confusion. All interviews were conducted in Arabic, the participants’ native language, audiotaped, and transcribed verbatim using Microsoft Word.

The data were analyzed thematically, a process that involves identifying, analyzing, organizing, describing, and reporting themes within a dataset [28]. Thematic analysis is an effective method for evaluating the perspectives of diverse study participants, highlighting similarities and differences, and revealing unexpected insights [29]. To interpret the data, I followed the six steps outlined by Braun and Clarke (2006) [28]: (a) familiarizing myself with the data; (b) generating initial codes; (c) searching for themes; (d) reviewing themes; (e) defining and naming themes; and (f) writing the report. I began by listening to the interview recordings and reading the transcripts to become familiar with the data. In the second phase, I employed both deductive and inductive descriptive coding approaches to analyze the transcripts. A list of codes was generated, refined, and organized into initial themes and subthemes. Subsequently, I examined the codes extracts for each theme and subtheme to ensure they cohered logically. Next, I evaluated each theme to determine which aspects of the data it captured and how these were relevant to my study. Once the themes were fully established, I proceeded with the final analysis and report writing. The data were analyzed in Arabic to capture deeper meanings from the transcripts, and the results were then translated into English.

To straighten the rigor of this study, various strategies were employed, focusing especially on credibility and transferability. Member checking was used to enhance credibility by asking participants during data collection about the meaning of their experiences and responses during the interviews to ensure that I accurately captured what they intended to convey. To support transferability, I provide a detailed description of the methods to allow others to assess the applicability of the findings to different contexts. Furthermore, to improve confirmability during data collection and analysis, I maintained reflective journals to record my thoughts, assumptions, and concerns throughout the research process, aiming to minimize potential bias. I also created an audit trail to document the decision-making process at each stage of the study.

## 3. Results

Several common themes emerged from the data, which were organized into three primary categories: (a) self-assessment of knowledge, (b) role toward dyslexic cases, and (c) the perceived needs for effectively working with dyslexia cases. One of these themes includes subthemes.

### 3.1. Theme One: Self-Assessment of Knowledge

All school-based SLPs who participated in the study indicated that they either have no knowledge or very limited knowledge about dyslexia. Some statements reflecting this included: “Honestly, I don’t know much about them” (p. 5) and “I know very little about dyslexia; I watched a ten-minute video about it when you told me about the purpose of this study” (p. 4). Several participants also asserted that the majority of SLPs lack knowledge about dyslexia. One stated, “Unfortunately, I think that almost all school-based speech therapists have no knowledge or understanding of something called dyslexia” (p. 1). In contrast, some participants held misconceptions about dyslexia. Participant 3 claimed that remote learning could cause dyslexia, saying, “Most students now have dyslexia, especially after the coronavirus pandemic. Dyslexia has become more prevalent among them due to the shift to distance learning. This has contributed to its increased prevalence.” Another participant indicated that dyslexia means reading words in reverse, stating, “I know some information about dyslexia; it is when students read backward” (p. 1).

Interestingly, multiple participants felt that identifying students with dyslexia could be easier than remediating them, as illustrated by the following statements: “I mean, sometimes I can tell that kid has dyslexia, but I do not know how to resolve the problem” (p. 3), and one stated, “Honestly, if you bring me a student and ask me to diagnose him, I might discover that this student has dyslexia, but I cannot determine an appropriate treatment method” (p. 1).

### 3.2. Theme Two: Role Towered Dyslexia Cases

The majority of participants expressed the belief that they do not hold responsibility for dyslexic cases. They indicated that dyslexia is not a speech disorder and should be addressed by special education teachers, specifically those working with students with learning disabilities. Participant 4 mentioned, “Based on my knowledge, teachers specialized in learning disabilities are the ones responsible for developing educational plans for students with dyslexia.” Furthermore, Participant 1 stated that:

“Dyslexia is not classified as a speech and language disorder. Speech disorders typically refer to speech impediments, voice disorders, and slurred speech. Yah, dyslexia does not fall under these categories; it is considered outside this specialty”.

(p. 1)

Some participants claimed that they are not legally responsible for providing services to students with dyslexia. Participant 2 noted that her role, based on her previous training and the school system, was limited to serving students from special education classes.

“Dyslexia is always addressed by teachers specialized in learning disabilities. This is what we have learned and studied previously. I only work in the field of special education, and most of the cases I handle involve hearing impairments. According to school policies, we are not permitted to take students from regular public school classes, even if they have dyslexia. Instead, they are always assigned to teachers specialized in learning disabilities”.

(p. 2)

Participant 5 also commented on the same issue, saying, “We refer dyslexia cases to the learning disability teachers because they are the specialists in this matter according to the education policy.”

Despite their perceived role in addressing dyslexia, some participants maintained a positive outlook on their responsibilities toward students with dyslexia. Several participants believed that their role could be secondary and supplementary to that of special education teachers. Participant 2 stated, “I believe I could work in the area of reading fluency or pronunciation with those kids.” The same view was expressed by Participant 4, who said:

“It’s possible that we both have roles, which will be shared between me and the teacher. For example, correcting pronunciation is very important for fluency in speech. The student should know how to spell and read the word. I feel that these aspects are interconnected, and my work complements the teacher’s.”

### 3.3. Theme Three: The Perceived Needs for Effectively Working with Dyslexia Cases

Another significant theme identified was the anticipated needs to effectively address the educational requirements of students with dyslexia. All participants agreed that several factors could play a crucial role in helping them identify and remediate dyslexia. Three subthemes or factors were identified within this main theme: (a) professional development, (b) collaboration with others, and (c) administrative support.

#### 3.3.1. Professional Development

Participants reported a significant need for training to effectively work with students with dyslexia, citing a lack of training at both the pre-service and in-service levels. Nearly all participants noted that they did not receive any dyslexia-related training during their pre-service education: “In college, we didn’t learn anything about dyslexia. We might have come across a chapter in a book, but I really don’t remember” (p. 5), and “dyslexia… we didn’t study anything about it” (p. 1). This issue persisted at the in-service level, where participants also indicated that they had not received any training related to dyslexia or their roles as school-based SLPs since graduation. Some statements reflecting this were: “I have not received any dyslexia training or other professional development opportunities since I was hired” (p. 1) and “we do not have professional development opportunities at all” (p. 3). Additionally, two participants highlighted the general lack of resources and professional development opportunities in the field of speech and language pathology: “In the field of speech and language therapy, training programs are generally very few, and knowledge resources are also scarce” (p. 2), and “Unfortunately, training sessions in this field are very limited and rare” (p. 1).

#### 3.3.2. Collaborating with Others

The majority of participants recognized the importance of collaboration with other school professionals, such as general education teachers, special education teachers, and parents, when working with students with dyslexia. Some statements to this effect were “The entire team must work toward a single goal, not just the learning disabilities teacher. This means that parents play a major role, as do the classroom teacher and the speech and language teacher” (p. 2), and “A situation where there is reading difficulty requires the regular classroom teacher to collaborate with the learning disabilities teacher and also with a speech and language disorders specialist” (p. 5).

#### 3.3.3. Administrative Support

Participants reported the absence of school policies clearly defining their responsibilities in supporting students with dyslexia, which may hinder their ability to effectively assist these students. For example, one participant stated, “We are not allowed to take students from regular public school classes, even if they have dyslexia. They are always assigned to learning disability teachers” (p. 2). Another noted, “We refer dyslexia cases to the learning disability teachers because they are the specialists in this matter according to education policy” (p. 5). Additionally, some participants highlighted time constraints caused by staff shortages and heavy workloads: “I don’t want to shock you, but I’m the only specialist in this county” (p. 2), and “Due to the large number of cases, neither time nor schedule allows me to take cases from general education classes” (p. 4).

## 4. Discussion

The aim of this study was to explore school-based SLPs’ perceptions of their ability to identify and remediate students with dyslexia.

### 4.1. Self-Assessment of Knowledge

The participants reported little to no knowledge about dyslexia. These findings are consistent with previous studies conducted worldwide. For example, a study in South Africa by Geertsema and Le Roux (2020) found that the majority of SLPs lacked competence in managing dyslexia [30]. Similarly, a study in the USA by Loveall et al. (2022), involving 271 SLPs, examined their perspectives on the scope of practice related to reading difficulties [31]. The SLPs expressed a lack of confidence in their ability to diagnose and treat reading disorders.

It was also evident from the interviews that some participants held misconceptions about dyslexia. They believed that dyslexia involves reading words backward and that it could result from online teaching and learning. This finding aligns with existing literature on common misconceptions about dyslexia among school professionals, which include word reversals [17,18,32] and a literacy-poor home environment [22]. Enhancing participants’ knowledge can correct these misconceptions by providing accurate information, leading to more effective assessment and remediation practices.

### 4.2. Role Toward Dyslexia Cases

The majority of participants indicated that they do not have a role in identifying or supporting individuals with dyslexia. Several participants also stated that they did not perceive themselves as legally responsible for providing services to students with dyslexia. However, The Ministry of Education (2025) stated that the responsibilities of school-based SLPs involve evaluating and treating students who have language disorders [21]. This belief suggests a potential knowledge gap or variability in the perceived scope of professional duties and legal obligations related to dyslexia support within educational settings, which could have practical implications for access to services.

Conversely, some participants viewed their role as secondary to that of special education teachers in supporting students with dyslexia. These findings were consistent with international literature. According to an American national study involving over 600 school-based speech–language pathologists, 50% believed literacy should not be included within their scope of practice [33]. Similarly, a recent study by Loveall et al. (2022) reported that SLPs perceived their role as secondary in assessing and remediating reading problems [31]. Thus, this perception may limit their engagement in essential interventions and support for students with dyslexia.

The lack of dyslexia-specific training may have shaped clinicians’ understanding of what falls within their roles and responsibilities. Ehren and Ehren (2001) indicated that SLPs may be hesitant to provide reading-focused therapy due to insufficient expertise and training in this area [34]. Furthermore, Hogan (2018) suggested that while SLPs generally feel confident in their ability to assess, treat, and consult on written text comprehension, they often lack the same confidence when it comes to assessing, treating, or consulting on word reading and dyslexia [19]. Another potential reason for their viewpoints could be administrative policies that discourage SLPs from working with students with dyslexia due to an unclear definition of the SLPs’ scope of practice regarding dyslexia. This factor has previously been identified as a barrier to their involvement in the USA and Australia [35,36].

### 4.3. The Perceived Needs for Effectively Working with Dyslexia Cases

Regarding perceived needs, school-based SLPs acknowledged that a lack of knowledge and skills related to dyslexia poses a significant challenge when working with students affected by this condition. They reported receiving no specific training on dyslexia at either the pre-service or in-service levels. They emphasized the importance of professional development opportunities to enhance their ability to support these students. Internationally, the need to improve school-based SLPs’ knowledge of dyslexia is widely recognized as a crucial factor in providing more effective evaluations and intervention services [30,31,32,37]. Ideally, SLPs should have access to comprehensive training on dyslexia during their pre-service education, as well as ongoing professional development at the in-service level, to be well-prepared to assist students experiencing reading difficulties in school settings.

Additionally, collaboration with teachers was identified as a crucial factor for SLPs to work effectively with students who have dyslexia. Teachers shared this view, emphasizing the importance of such collaboration as well. According to Watson et al. (2020), teachers in settings where SLPs are present report valuing their support in providing reading intervention services [38]. This suggests that teachers also recognize the significance of teamwork. Effective inclusive education, according to Snowling et al. (2022), is built on collaboration between teachers and speech–language pathologists, particularly during the early school years when essential reading skills are being developed [39].

Lastly, participants stressed the importance of administrative support through the enactment of policies that clearly define the roles of SLPs in schools, particularly regarding support for students with dyslexia. The absence of clearly defined school-role expectations has been widely recognized in the literature as a barrier to SLP involvement in such cases. Alves (2021) noted a lack of consistency in the detection and remediation processes for dyslexia across states in the USA, highlighting that even in areas with universal screening programs, it is often unclear who is responsible for these procedures [40]. In Australia, Stephenson et al. (2024) suggested that a lack of awareness regarding the scope of practice of SLPs may result in missed opportunities for collaborative practices [36]. In line with these observations, Watson et al. (2020) argued that while written language intervention falls within the scope of practice for SLPs, those working in school settings may need to advocate for their involvement in written language instruction [38]. Thus, although school-based SLPs are considered crucial in recognizing, diagnosing, and supporting students with dyslexia (ASHA, 2001), more precise guidelines regarding their role within addressing reading difficulties in educational settings are needed [41]. Thus, schools should develop formal role agreements that clearly delineate the responsibilities of SLPs in dyslexia identification and remediation, distinguishing them from, yet complementing, the roles of reading specialists.

## 5. Limitations and Future Directions

This study has several potential limitations that should be considered when interpreting the findings. First, all participants were from the same geographical region and attended public schools, which may limit the generalizability of the findings to other areas with different policies, educational contexts, and resource allocations. Variations in practices and access to training across regions and school systems could result in differing levels of dyslexia-related knowledge and support needs among SLPs. Second, the study was limited by a relatively homogeneous sample, consisting exclusively of SLPs with a minimum of six years of experience in school settings. Including novice SLPs would provide a more diverse sample and potentially yield additional insights into the research topic.

## 6. Conclusions

The findings of this study closely align with results observed in other countries, highlighting a broader, consistent pattern in the understanding of dyslexia among school-based SLPs across different educational systems. Most participants expressed misconceptions about dyslexia and rated their knowledge of the condition as either very poor or nonexistent. Regarding the management of reading difficulties, school-based SLPs often perceived their role as minimal, with some considering it secondary to that of special education teachers. Participants emphasized the need for comprehensive professional development opportunities aimed at enhancing their knowledge and proficiency in this field. Furthermore, they underlined the importance of fostering effective collaboration with teachers. Participants also noted that to facilitate these changes, strong administrative support is essential, including the establishment of clear policies and guidelines defining their scope of practice in managing dyslexia.

## Figures and Tables

**Table 1:** Participant Demographic Information.

P ^#^	Gender	Highest Academic Qualification	Years of Experience	Classroom Type
1	Male	Bachelor’s degree	8	Public schools, Self-contained classrooms Special education center
2	Female	Bachelor’s degree	10	Public schools, Self-contained classrooms, Special education schools
3	Male	Bachelor’s degree	9	Public schools, Self-contained classrooms, Non-government organization
4	Female	Bachelor’s degree	7	Self-contained classrooms
5	Male	Bachelor’s degree	10	Public schools, Self-contained classrooms, Special education center
6	Male	Diploma	6	Special education schools

**^#^** P = Participant.

## Data Availability

The datasets presented in this article are not readily available because the data are part of an ongoing study.

## Abbreviations

The following abbreviations are used in this manuscript:

SLP	speech–language pathologist
SPA	Saudi Press Agency
APA	American Psychiatric Association
ASHA	American Speech–Language–Hearing Association
